# Risks and benefits of TIPS in HCC and other liver malignancies: a literature review

**DOI:** 10.1186/s12876-023-03047-0

**Published:** 2023-11-20

**Authors:** Anna Fichtl, Thomas Seufferlein, Eugen Zizer

**Affiliations:** Department of Gastroenterology and Hepatology Internal Medicine I, University Ulm, University Hospital Ulm, Albert-Einstein-Allee 23, Ulm, 89081 Germany

**Keywords:** Transjugular intrahepatic portosystemic shunt (TIPS), Clinically significant portal Hypertension (CSPH), Hepatocellular carcinoma (HCC), Liver metastases, Liver Cirrhosis

## Abstract

**Background:**

Transjugular intrahepatic portosystemic shunt (TIPS) is a well-validated treatment option for clinically significant portal hypertension (CSPH) in the context of liver cirrhosis. Its high efficacy and safety in the management of treatment-refractory ascites and variceal bleeding have been extensively proven. Contraindications for TIPS include severe right heart failure, hepatic encephalopathy, and sepsis. However, the role of liver malignancy in TIPS is debatable. Mostly, primary liver malignancies such as hepatocellular carcinoma (HCC) emerge from advanced liver diseases. Coexisting portal hypertension in HCC often results in limited treatment options and a poor prognosis.

**Summary:**

Previous studies have shown that TIPS implantation in patients with HCC is technically feasible and is usually not associated with major adverse events. Furthermore, TIPS may help in bridging the time to liver transplantation in early HCC and allow for locoregional treatment in advanced HCC. However, several studies suggest that seeding tumour cells to the lungs by TIPS placement might worsen the prognosis.

**Conclusions:**

TIPS placement in patients with coexisting liver malignancy remains a case-by-case decision, and there is no profound evidence allowing general recommendations. This review aims to provide a state-of-the-art overview of the potential risks and benefits of TIPS placement in patients with liver malignancies.

## Background

Chronic liver diseases are often complicated by the development of ascites, variceal bleeding and other symptoms of portal hypertension. Besides systemic treatment, e.g., with non-selective beta-blockers, transjugular intrahepatic portosystemic shunt (TIPS) is effective for managing clinically significant portal hypertension (CSPH) [1]. Following the Baveno VII Consensus Conference recommendations, TIPS is a valuable treatment option in variceal rebleeding, treatment-refractory ascites, Budd-Chiari syndrome, portal vein thrombosis without cirrhosis and thrombosis of the portal vein trunk in cirrhosis without recanalization on anticoagulation [2]. Contraindications for TIPS in patients include severe heart and liver failure (Child–Pugh score of more than 13 or a Model for End-stage Liver Disease (MELD) score of more than 19) and chronic or recurrent hepatic encephalopathy (HE) [3]. According to a review article from 2022, hepatocellular carcinoma (HCC) is the third most prominent contributor to cancer-related deaths globally [4]. In over 90% of cases, HCC is diagnosed in the setting of chronic liver diseases [5].

Consequently, patients with HCC often suffer from CSPH and may benefit from TIPS implantation. However, the role of TIPS in HCC is under debate due to the risk of liver failure, HCC spread and potentially less effective subsequent locoregional HCC treatment [6]. Most randomized controlled trials (RCT) investigating TIPS and high-volume paracentesis excluded patients with HCC [7–11]. Thus, the role of TIPS in HCC is not well-studied. Even less evidence exists regarding the role of TIPS in metastatic liver disease.

This review summarizes the current knowledge about the risks and benefits of TIPS placement in patients with liver cirrhosis complicated by liver malignancies.

## Main text

### Methods

A literature search was performed in PubMed and ScienceDirect databases. The search strategy included the keywords “HCC”, “hepatocellular carcinoma”, “hepatic cancer”, “transjugular portosystemic intrahepatic shunt”, “TIPS”, “liver malignancy”, “liver metastases”, “malignant” and “pseudocirrhosis”. Articles containing these keywords, if available online and written in English, were included in this review. Additionally, related articles listed in the PubMed sections “similar articles” and “cited by” were reviewed and suitable articles were included.

### TIPS in patients with HCC

#### Feasibility and efficacy

Several retrospective reports have demonstrated the feasibility of TIPS implantation in patients with HCC: The success rate in several retrospective studies including a total of 496 patients was more than 95% [12–17]. While there is no established standard for measuring clinical response, several trials reported a high rate of partial and complete remission of portal hypertension (PH) symptoms after TIPS implantation [13–15, 18]. The median overall survival (OS) of patients with HCC who received TIPS was heterogeneous, presumably due to differences in patient eligibility, predominant symptoms of PH, stages of liver disease (Child-Pugh) and HCC (Barcelona Clinic Liver Cancer Staging, BCLC) (Table 1). The rate of shunt dysfunction and consequently required TIPS revisions also varied considerably among the retrospective studies: During the follow-up, shunt dysfunctions were observed in 7.1% [15] to 20.7% [13]. TIPS revisions were required in 7.1% [15] to 15.5% [13]. Those differences among the studies can be attributed, as discussed similarly to OS, to varying patient characteristics and different sample sizes, as well as differing techniques and stents used for TIPS placement.

**Table 1:** Retrospective trials enrolling patients with HCC who received TIPS placement

Author	Main exclusion criteria	Enrolled patients with HCC + TIPS (n)	Pre-TIPS C-P stage (A/B/C)	BCLC (A/B/C/D)	Clinical response of CSPH to TIPS (partial/complete remission) (%)	Median survival (months)
**Yan H, et al. 2022** [18]	Tumour volume > 70% of the liver, primary CCC, multiple liver cysts, liver failure, severe cardio-pulmonary dysfunction, biliary and pancreatic obstruction	123	32/79/12	14/27/71/11	18.7/74.8	10.7
**Qiu Z, et al. 2022** [15].	Tumour volume > 70% of the liver, lung/bone metastases, multiple liver cysts, congestive heart failure, sepsis, moderate/severe pulmonary hypertension, biliary obstruction, severe coagulopathy	42	12/26/4	0/0/38/4	20/72	9.6
**Luo S., et al. 2019** [16]	Portal vein thrombosis, history of HE, severe right-sided heart failure, polycystic liver disease, dilated biliary ducts, age > 75 years, bilirubin > 5 mg/dL, creatinine > 3 mg/dL, C–P score > 11, MELD score > 18, sepsis, spontaneous bacterial peritonitis, patients who had undergone transplantation	217	54/129/34	18/107/53/34	NA	50
**Liu L., et al., 2014** [13]	Treatments for CSPH other than TIPS, no PVTT	58	11/34/13	0/0/35/23	15/80	2.5
**Bettinger D, et al. 2015** [14]	Intra– or extrahepatic cholangiocarcinoma or metastases from extrahepatic malignancies	40	3/29/8	NA	74 and 100^1^	6.1

^1^Clinical response to TIPS in 74% of patients treated for ascites and in 100% of patients treated for variceal bleeding. HCC, hepatocellular carcinoma; TIPS, transjugular intrahepatic portosystemic shunt; C-P, Child-Pugh; CSPH. clinically significant portal hypertension; CCC, cholangiocellular carcinoma; HE, hepatic encephalopathy; PVTT, portal vein tumour thrombosis; MELD, Model for End-stage Liver Disease; NA, not available

Several RCTs [19, 20] and observational studies [21–23] investigating TIPS in patients with cirrhosis without HCC confirmed a survival benefit of pre-emptive TIPS for patients with Child-Pugh B or C in combination with active bleeding. Patients with HCC and CSPH benefitted from TIPS placement and showed a longer survival time: in two observational studies, the outcome of patients with HCC and CSPH (n = 437 in total) was analyzed [15, 16]. In both studies patients with HCC and refractory ascites, hydrothorax or variceal bleeding were enrolled and subjected to either TIPS placement and palliative HCC treatment (Group A; n = 259 in total) or standard of care consisting of drug therapy of PH and palliative HCC treatment (Group B; n = 178 in total). In the more recent study, matched patients were allocated 1:1 to Group A (n = 42) and Group B (n = 42). Here, the control rates of variceal rebleeding and ascites were significantly better (p < 0.001, respectively) in Group A [15]: 18 out of 25 patients suffering from refractory ascites or hydrothorax in Group A achieved complete remission, but none of the 25 patients with refractory ascites or hydrothorax in Group B. A variceal re-bleeding occurred in 1 out of 20 (Group A) versus in 5 out of 19 (Group B) patients with prior variceal bleeding. In the older study, patients with similar baseline characteristics were also allocated to either Group A (n = 217) and Group B (n = 136). Notably, patients receiving Sorafenib were excluded in this study, unlike the other study. The absorption of unresponsive ascites within one month (39 of 44 cases in Group A vs. 9 of 22 cases in Group B), the recurrence of variceal bleeding (28 of 168 cases in Group A vs. 56 of 114 cases in Group B), and the recurrence of unresponsive ascites (13 of 44 cases in Group A vs. 19 of 22 cases in Group B) showed notable variations between the groups in favor of Group A (with p-values of 0.017, 0.023, and 0.009, respectively) [16].

Altogether, patients having received TIPS survived significantly longer than patients without TIPS according to the results of the last two mentioned studies: the larger study’s observation period concluded at 5 years, during which 88 patients (41.51% out of 212) in Group A and 22 patients (16.18% out of 136) in Group B had survived (median survival: 50 versus 33 months, p = 0.006) [16]. In the smaller study comprising a total of 84 patients who simultaneously suffered from portal vein tumour thrombosis (PVTT) Group A showed a significantly better median OS compared to Group B (9.6 [95% Confidence Interval: 7.1, 12.0] vs. 4.9 [95% Confidence Interval: 3.9, 5.8], months, p < 0.001) [15]. However, there are still no prospective studies confirming a potential survival benefit from TIPS-insertion in these patients.

### TIPS for variceal bleeding in HCC

HCC patients with liver cirrhosis and variceal bleeding have a significantly poorer prognosis than HCC patients without bleeding [24] or patients with variceal bleeding but without HCC [25]. According to a case-control study comparing the outcome after variceal bleeding in 146 patients with and without HCC, secondary relapse prevention by systemic treatment with vasoactive drugs or endoscopic band ligation led to a survival benefit in both groups, but the rebleeding rates and the mortality in patients with HCC were significantly higher than in those without HCC (log-rank test p = 0.001, p < 0.001, respectively) [26]. It is well known that TIPS can reduce recurrent variceal bleeding rates and improve survival in patients with cirrhosis without HCC [19, 27, 28]. However, its potential to prevent variceal bleeding in liver cirrhosis in the context of concomitant HCC is less well-understood.

The first cases of patients suffering from variceal bleeding associated with hepatic neoplasia and TIPS placement were published in the 1990s, when a TIPS was successfully implanted in a 53-year-old man with colorectal cancer and liver metastases and in a 41-year-old man with HCC: in both cases TIPS intervention was performed as a palliative procedure for refractory variceal bleeding [29, 30]. A recent RCT investigated the benefits of TIPS in HCC patients on active therapy with tyrosine kinase inhibitors (TKI). 106 patients were randomized (1:1) to receive either TIPS placement or endoscopic/ß-blocker therapy. 14 of 54 patients in the conventional therapy group had a rebleeding episode, but only three of 52 TIPS patients were affected by rebleeding (p < 0.001) during a median follow-up of 16 months. Furthermore, also secondary endpoints were superior in the TIPS intervention group (OS after 18 months: 73% vs. 15% (p < 0.001), improvement of Child-Pugh score: 76% vs. 15% (p < 0.001), maintenance of TKI at full dose: 51.9% vs. 7.4% (p < 0.001), fewer TKI discontinuations: 14.8% vs. 46.3% (p < 0.001)). Thus, this study demonstrated that TIPS in HCC with variceal bleeding is not only feasible but also leads to improved OS, higher TKI treatment adherence and lower rates of therapy interruption [31]. A multicentre observational trial investigating TIPS implantation to prevent gastric variceal rebleeding in HCC patients reported similar findings [17]. More importantly, patients with both a high MELD score (> 15 pts.) and an advanced liver cancer stage (BCLC C–D) had an increased baseline risk of 6-week mortality after acute variceal bleeding without intervention [25]. Such high-risk patients may substantially benefit from TIPS implantation. In summary, TIPS placement for acute variceal bleeding in the setting of HCC is supported by current data.

### TIPS for refractory ascites or hydrothorax in HCC

There is evidence that TIPS can sufficiently control ascites in patients with HCC. A multicentre study evaluating the efficacy of TIPS in HCC and CSPH included 51 patients with variceal bleeding, 49 patients with refractory ascites and 23 patients with both variceal bleeding and refractory ascites. A control rate of 87% was achieved for refractory ascites due to TIPS, contributing to a significant improvement of the median Child-Pugh scores from 8 to 6 pts (p < 0.001) [18]. These findings were supported by two other, smaller studies reporting a response of ascites to TIPS in 20/27 patients (74.1%) [14], and in 19/20 patients (95%) [13].

In the setting of liver cirrhosis without HCC, TIPS is judged to be equally effective for refractory ascites and hydrothorax [32]. In a retrospective trial enrolling 73 patients with end-stage liver cirrhosis and hydrothorax, but without HCC, TIPS placement succeeded technically in all the patients, and a clinical response was shown in 79% (58/73) of the patients enrolled one month after TIPS placement and in 75% (30/40) 6 months after the TIPS intervention. The median survival among clinical responders within 1 month was 728 days (with a range of 320 to 1,135), while among nonresponders, it was 85 days (with a range of 0 to 209) (p = 0.001) [33]. Hepatic hydrothorax is a rather rare complication of cirrhosis, so there is only little data about TIPS in patients with hydrothorax and HCC. In a retrospective trial including eight patients with hydrothorax the latter was decreased in all patients after TIPS, but it is not clear whether further thoracocenteses were necessary [13]. Another retrospective study investigating the efficacy of TIPS in patients with HCC, PVTT and CSPH enrolled 50 patients with refractory ascites and/or hydrothorax [15]. 25 patients received a TIPS procedure resulting in a complete remission of ascites and/or hydrothorax in 72.0% (18/25) and a partial remission in 20.0% (5/25) during follow-up until death. In comparison, another 25 patients with similar baseline characteristics received conservative treatment of their CSPH, leading to a complete remission in 0% and a partial remission in 28.0% (7/25) of cases (p < 0.001). ln the context of this study, complete remission was defined as the complete disappearance of ascites and/or hydrothorax, while partial remission referred to the presence of ascites and/or hydrothorax without the need to be punctured. An interpretation of these data for hydrothorax is not possible, as the authors did not report separate data on hydrothorax, presumably due to the low number of subjects.

Altogether, TIPS placement is technically feasible and efficient in patients with HCC and refractory ascites, while more data is needed concerning TIPS in patients with refractory hydrothorax.

### TIPS for PVTT in HCC

PVTT is associated with a higher risk of variceal bleeding [34] and a poor prognosis in patients with HCC [35]. The success rate of TIPS implantation in patients with HCC and PVTT varies from 68.8 to 100% [13, 36–38]. TIPS has proved to be effective in treating ascites, bleeding, and diarrhea because of PVTT [37]. TIPS patency one year after placement was observed in 100% of 11 cases in a small observational study [36]. and in 95% of 52 patients in an RCT [31]. To prevent stent occlusion due to tumour invasion, the stent should penetrate the complete thrombotic mass [37].

A case-control study demonstrated significantly longer survival (9.6 vs. 4.9 months) in 42 patients with HCC and PVTT receiving TIPS compared to a matched cohort of 42 patients with HCC and PVTT without TIPS (p < 0.001) [15]. However, the different survival rates might also be due to the fact that a sequential anti-HCC therapy with TKI was performed in all the patients receiving TIPS, while patients without a TIPS procedure received only symptomatic and supportive treatment, such as vasoactive drugs, antibiotics, and endoscopic treatment. TIPS-related complications were observed in 21.6% of cases including one patient with intraperitoneal bleeding. Several trials enrolling patients with both HCC and PVTT equally reported severe TIPS-related complications: in a retrospective study 2/95 patients died of intra-abdominal bleeding or thoracic cavity bleeding during percutaneous intrahepatic puncture [38]. Another study observed tumour rupture in 5/58 patients [13]. This high rate of adverse effects may be due to the high number of patients with complete rather than partial PVTT and with arteriovenous fistula and portal cavernoma, respectively. Generally, the outcome of patients undergoing TIPS for PVTT is highly influenced by the degree of PVTT and by the severity of liver disease [15].

### Post-TIPS hepatic encephalopathy and procedure-related complications

According to a systematic review including five case series with a total of 280 patients with HCC undergoing a TIPS procedure, the rates of major TIPS-related complications were similar to those reported for patients without HCC (accelerated liver failure: 3%; death: 1%), suggesting that TIPS placement may be as safe in patients with HCC as in patients without HCC [39]. The major causes of death in patients with HCC receiving TIPS reported in the literature are tumour progression [14–16, 31] and variceal rebleeding [13, 40].

In two studies, a transient, significant rise in liver enzymes post TIPS is described in 2/42 (4,8%) and 1/58 (1,7%) of patients [13, 15]. In all three cases, liver enzymes spontaneously decreased after a few days on conservative treatment. The reported rate of liver failure together with multiple organ failure after TIPS placement ranges from 0% to 7,7% [6, 13, 14, 18, 40].

Tumour rupture is a rare but perilous complication of HCC. It seems to occur more frequently in patients with vascular injury and PVTT [40]. However, apart from one report [13], there is little evidence of a frequent occurrence of this severe complication.

Besides the procedure-related complications of TIPS, there is a remarkable risk of post-TIPS HE since the shunt bypasses nutrient-rich blood flowing directly to the liver veins, leading to a lower rate of elimination of ammonia in the blood [41]. In 2015, two retrospective trials demonstrated the development of HE in 40% of patients with HCC after TIPS insertion [14, 42]. Subsequent studies reported fewer episodes of post-TIPS HE [6, 15–18], possibly due to improvements in the TIPS technique and a selection of less morbid patients. Regarding the degree of hepatic encephalopathy (HE), there is heterogeneous reporting among different studies. Three studies only indicate the presence or absence of HE [16, 17, 42], whereas other studies explicitly mention the grade of HE: Bettinger et al., Qiu et al., and Yan et al. reported that after TIPS placement, HE grades 1–2 were predominant [14, 15, 18]. In Bettinger et al.‘s study, HE grades 1–2 were observed in 15 out of 40 patients (37.5% of all TIPS patients) [14], in Qiu et al.‘s study, it was 4 out of 42 patients (9.5% of all TIPS patients) [15], and in Yan et al.‘s study, it was 12 out of 123 patients (9.8% of all TIPS patients) [18]. HE grades 3–4 in patients having received TIPS were rare, happening in 1 out of 40 patients (2.5% of all TIPS patients) in Bettinger et al.‘s study [14], 1 out of 42 patients (2.4% of all TIPS patients) in Qiu et al.‘s study [15], and 1 out of 123 patients (0.8% of all TIPS patients) in Yan et al.‘s study [18].

In a retrospective study investigating risk factors for post-TIPS HE in 279 HCC patients, 114 patients developed HE three months after TIPS placement [42]. The study revealed a high pre-TIPS MELD score (14.7 in the HE-group vs. 9.8 in the non-HE group), a number of more than three transarterial chemoembolization/transcatheter arterial embolizations (TACEs/TAEs) performed post TIPS placement and a high decrease in the portosystemic pressure gradient as main risk factors for HE after TIPS. Furthermore, impaired liver function and sarcopenia were identified as predictive factors for an episode of HE post TIPS [43, 44]: a pre-TIPS Child–Pugh score > 10 and a MELD score > 15 [45] as well as radiologically confirmed muscle waste [46] increased the risk of HE after TIPS placement. Since the prevalence of sarcopenia (39%) is especially high in patients with HCC [47], the latter might be even more relevant for post-TIPS HE in patients with HCC than in patients with cirrhosis without HCC.

Ultimately, many patients with HCC and CSPH undergo anti-tumour treatment before or after TIPS implantation. Since anti-tumour treatment itself can cause severe adverse events, some of them similar to those observed after TIPS placement, the evaluation of purely TIPS-related complications can be challenging.

### Impact on local anti-tumour treatments

The combination of TIPS with local HCC treatment is rarely reported. In a retrospective study investigating the treatment-related outcome in a total of 80 patients with HCC and variceal bleeding, TIPS combined with TACE was superior to endoscopic intervention, consisting of ligation and sclerosing agent injection, combined with TACE [48]. In the group with TIPS and TACE combined (n = 42), a significantly lower rebleeding rate (7.14% vs. 15.79%, p = 0.025) and longer progression-free (4.55 vs. 2.50 months, p < 0.001) and OS (13.75 vs. 8.50 months, p = 0.0058) were observed compared with the group in which the endoscopic procedure was combined with TACE (n = 38). The incidence of HE after TIPS (4.8%) or after endoscopic intervention (5.3%) did not significantly differ between the two groups.

In contrast, TACE is also known to cause hepatic ischemia [49]. Hence, the liver perfusion per se can be further reduced by TIPS, which diverts the inflow of the portal vein to the systemic circulation [50], thereby potentially resulting in an insufficient perfusion of the liver itself. In fact, there is evidence of increased ischemic hepatotoxicity [51–53] and impaired treatment response to TACE [53] in patients with previously implanted TIPS compared to patients without TIPS. However, a systematic review of six studies, including 536 patients with HCC showed that the response rate of TACE was lower in patients with TIPS compared to TACE alone, but this effect was not statistically significant (p = 0.171) [54]. In a systematic review of 21,461 patients receiving TACE the risk of liver failure after TACE was 2.9% [55]. Therefore, liver enzyme levels and liver synthesis parameters should be monitored carefully during and after TACE, especially in the context of a potentially planned TIPS placement following TACE. Interestingly, the use of TACE with drug-eluting beads instead of conventional TACE was associated with fewer hepatotoxic effects (hepatic failure after 30 days: p = 0.027) and resulted in improved OS (11.4 vs. 9.1 months, p < 0.001) after TIPS [56]. However, according to data from a multicentre retrospective trial, TACE with drug-eluting beads performed in 394 patients compared to conventional TACE performed in 608 patients caused more hepatobiliary injury in terms of bile duct dilation (15.5% vs. 7.5%, p < 0.001), portal vein narrowing (4.6% vs. 1.6%, p = 0.006) and cholecystitis (3.0% vs. 2.0%, p = 0.28) [57]. Other locoregional treatments such as thermal ablation may result in even fewer complications than TACE [58] and thereby make patients with CSPH more readily eligible for TIPS.

Then again, for patients with CSPH requiring systemic HCC treatment, TIPS implantation can be favoured in certain circumstances: By relieving the symptoms of PH, TIPS renders the patients eligible for further HCC treatment [12, 16, 59]. In conclusion, the optimal timing for TIPS and/or local therapy approaches in HCC should be determined individually based on the disease stage, tumor characteristics, and patient’s condition.

### De novo HCC and HCC spreading after TIPS implantation

There is some evidence of de novo HCCs caused by regenerative and dysplastic nodules after performance of a portosystemic shunt: in a post-mortem analysis from 1985, a higher prevalence of HCC was observed in male patients with surgical portosystemic shunts (relative risk of HCC for male patients with a survival of at least 6 months after shunt creation: 2.54, 95% Confidence Interval 1.27–5.05) [60]. In a retrospective study comprising a database analysis of 106 patients with cirrhosis who had received TIPS, previously unknown HCCs were diagnosed in a significant number of patients (n = 41) three months after TIPS implantation [61]. Another retrospective study investigated the presence of occult HCC in the explanted livers of patients undergoing liver transplantation due to variceal bleeding or refractory ascites. A total of 640 patients with HCC and/or cholangiocellular carcinoma were included in the study. For most of these patients (86%), HCC was already known prior to liver transplantation [62]. 40 of 640 patients in the trial underwent TIPS implantation prior to liver transplantation. Interestingly, with 80%, patients with TIPS were significantly more likely to present with an occult disease in the liver explants than patients in the non-TIPS group with 43% (p < 0.001). In the context of the study, an occult disease referred to an HCC lesion that was not visible in pre-transplant imaging. TIPS per se did not alter the disease-free or OS of this cohort. Furthermore, the presence of portal vein thrombosis correlated with occult HCC in the evaluated patients in a similarly pronounced way. It should be noted that the presence of TIPS and/or portal vein thrombosis may impair imaging quality. Consequently, it might be possible that in the case of a prior TIPS placement, preoperative imaging could lead to a falsely low detection rate of HCC lesions.

On the other hand, a case-control study among 1338 patients with cirrhosis, of whom 259 had had a TIPS procedure, found that the probability of developing HCC was significantly higher in the non-TIPS group (log-rank test: p = 0.002) [63]. Furthermore, the presence of TIPS possibly facilitates the detection of focal HCC-suspicious liver lesions via contrast-enhanced ultrasound by reinforcing arterial perfusion [64]. Altogether, the evidence of an association between TIPS and the de novo development of HCC tends to be low and is debatable [65].

In contrast to the issues discussed above, metastatic spreading of pre-existing HCC after TIPS placement could be a relevant issue due to the mechanical manipulation and the generation of a vascular short-cut. However, only few studies are investigating this point (Table 2). According to a systematic review of five studies published between 2003 and 2015 and including a total of 280 patients with HCC undergoing a TIPS procedure, the rate of lung metastases after TIPS placement was 1% (5 months (n = 1) and 72 months (n = 1) after TIPS placement) [39]. In comparison, an analysis of data retrieved from a database maintained by the US National Cancer Institute between 2010 and 2015 revealed a lung metastasis rate of 6.28% in 33,177 patients with HCC, accompanied by an annual incidence rate ranging from 0.9 to 1.1% [66]. Still, the incidence of lung metastases after TIPS procedure may be underestimated since there is no standardized periprocedural screening for lung metastases in patients undergoing TIPS placement [39].

**Table 2:** Clinical trials comprising patients with HCC and developing pulmonary metastases after TIPS placement

Author	Study type	Enrolled patients	Liver malignancy	Shunts traversing the tumour	Cases with pulmonary metastases post TIPS	Time between TIPS placement and diagnosis of pulmonary metastases (months)
**Tsauo J, et al. 2021** [17]	Retrospective, single-centre	126	HCC (n = 126)	8	3, amongst them no shunt traversing the tumour	3.9–32.9
**Zhao H, et al. 2018** [39]	Systematic review	280	HCC (n = 280)	35	2	5 and 72
**Wallace M, et al. 2003** [67]	Case series	9	HCC (n = 7) Pancreatic neuroendocrine metastases (n = 1) Metastatic gastrointestinal stroma tumour (n = 1)	9	2	5 and 10

TIPS: transjugular intrahepatic portosystemic shunt; HCC: hepatocellular carcinoma

Whether or not shunts traversing the tumour would increase the risk of lung metastases is unclear. There are two retrospective studies reporting on a TIPS placement through a known HCC lesion in the liver [17, 67]. In the larger study [17], TIPS was successfully placed through the malignancy in 8 out of 124 patients with HCC undergoing TIPS procedure. None of these 8 patients subsequently developed lung metastases. However, lung metastases after TIPS insertion were observed in 3 patients with HCC in whom TIPS had not been placed through the tumor. In the smaller study [67], TIPS was successfully placed through the tumor in 7 patients with HCC as well as in other 2 patients with liver metastases from extrahepatic malignancies. Among the HCC patients with TIPS traversing the tumour lung metastases occurred in 1 out of 7 cases [67].

### Consideration of tumour characteristics in HCC patients undergoing TIPS placement

Several retrospective and one prospective study provide information on key tumor characteristics such as size, location, and number of HCC lesions (Table 3).

**Table 3:** Clinical trials providing information on tumour characteristics of patients with HCC who received TIPS

Author	Enrolled patients with HCC + TIPS	Tumour size (cm)	Location	Single (1–2 lesions)	Multiple (> 3 lesions)
**Larrey E, et al. 2022** [6]	8	1.2–4.9	Central: 1 Non-central: 7	8	0
**Yan H, et al. 2022** [18]	123	NA	NA	42	81
**Qiu Z, et al. 2022** [15]	42	NA	NA	9	33
**Dong H, et al. 2021** [12]	13	NA	Central: 7 Non-central: 6	NA	NA
**Tsauo J, et al. 2021** [17]	126	Max. 4.2	NA	98	28
**Bettinger D, et al. 2015** [14]	40	1.6-8	Central: 15 Non-central: 25	21	19
**Liu L, et al. 2014** [13]	58	2.6–16	NA	38	20
**Yao J, et al. 2015** [42]	279	Max. > 5	NA	270	9
**Qiu B, et al. 2015** [78]	209	Max. > 5	NA	148	61

HCC: hepatocellular carcinoma; TIPS: Transjugular intrahepatic portosystemic shunt; NA: not available

Centrally located HCC lesions are situated, according to the traditional Coinaud definition, within liver segments IV, V, and VIII [68]. They are frequently connected to the liver’s vascular system [69], which often results in traversing the tumour during the TIPS procedure. In the limited number of studies where information on tumour location is provided [6, 12, 14], no clear evidence is given that TIPS placement for centrally located HCC lesions leads to more complications. However, larger prospective studies are necessary to confirm this. Similarly, more data are needed to facilitate recommendations for a potential cut-off regarding tumor size and single versus multiple tumor nodules in the liver for a planned TIPS implantation. Based on the available data, the tumour morphology of patients with HCC and CSPH should be individually and interdisciplinarily considered when evaluating the suitability for a TIPS placement.

### TIPS in patients with extrahepatic malignancies

TIPS is not routinely used in the setting of liver malignancies, especially not in liver malignancies beyond HCC. A case series of 38 patients with 41 liver malignancies including 13 cases of HCC, one case of cholangiocarcinoma, three cases of liver metastases and 22 cases of extrahepatic primary cancers was published in 2004 [70]. TIPS implantation was performed because of variceal bleeding (n = 16), transfusion-dependent portal gastropathy (n = 1), enlarging gastric varices prior to myelosuppressive treatment (n = 1), refractory ascites (n = 13), hepatic hydrothorax (n = 1) and Budd-Chiari syndrome (n = 3). The TIPS procedure was technically feasible (primary technical success in 97%) and safe in the patients enrolled, and there were no significant differences in OS between patients with intra- and extrahepatic malignancy.

More recently, one case report and one retrospective study described TIPS placement in a total of 12 patients with pseudocirrhosis [71, 72]. Pseudocirrhosis mimics clinical traits of cirrhosis but shows no signs of true cirrhosis in histological analysis [73]. While it is most common in breast cancer with metastatic liver disease [73, 74], there are only few published cases of pseudocirrhosis due to liver metastases in other tumour entities [75, 76]. In a case series of nine pseudocirrhosis patients (5 with colorectal cancer, 3 with neuroendocrine tumour, 1 with malignant hemangioendothelioma), TIPS intervention was performed because of variceal bleeding (n = 3) or ascites (n = 6) [72]. In eight of nine cases, TIPS placement was technically successful and led to a significant decrease in the portosystemic pressure gradient (p = 0.001); the symptoms of pseudocirrhosis were ameliorated in six of nine cases. In 6 of 9 patients mild to moderate HE (West Haven Grade 1–2) occurred post-interventionally. In the largest case series available on pseudocirrhosis, consisting of 120 female patients who suffered from pseudocirrhosis due to metastatic breast cancer, HE was observed at a rate of 9.2% (no TIPS placement had occurred in this context) [77].

Altogether, TIPS placement seems technically feasible in patients with liver malignancies other than HCC (Table 4), but more studies are needed in terms of safety and long-term outcome related to the procedure.

**Table 4:** Case series of patients with liver malignancies other than HCC who received TIPS placement

Author	Enrolled patients with liver malignancies + TIPS	Liver malignancy	Indication for TIPS	Technical success (%)	Post-TIPS HE (%)
**Shreve L, et al. 2022** [72]	9	Colorectal cancer (n = 5) NET (n = 3) malignant haemangioendothelioma (n = 1)	Acute variceal bleeding, refractory ascites	89	67
**Geeroms B, et al. 2018** [71]	3	Breast cancer (n = 3)	Refractory ascites	100	NA

HCC: Hepatocellular carcinoma; TIPS: Transjugular intrahepatic portosystemic shunt; HE: hepatic encephalopathy; NET: neuroendocrine tumour; NA: not available

## Conclusions

TIPS intervention in the context of HCC has the potential to attenuate CSPH in a significant way. Referring to recent studies, the rates of TIPS-related complications and post-TIPS HE in patients with HCC are similar to the rate of adverse effects in patients without HCC. Consequently, TIPS implantation in patients with HCC should be generally considered as a treatment option. Since there are no RCTs on this subject, the careful selection of patients and an individual risk-benefit analysis are mandatory. Attention should be paid to the risk of liver failure after TIPS in HCC, especially when combined with locoregional anti-tumour treatments such as TACE. There is no clear association between TIPS placement and the de novo development of HCC. More studies evaluating the risk of lung metastasis after TIPS intervention are needed to clarify the risk and relevance of TIPS-induced tumour spreading. Concerning the safety and efficacy of TIPS in patients with CSPH and liver malignancy other than HCC, there is little data, and no clear recommendations can be given for this group of patients.

## Data Availability

Datasets and materials used during the present study are available from the corresponding author upon reasonable request.

## Abbreviations

BCLC: Barcelona Clinic Liver Cancer Staging
CSPH: Clinically significant portal hypertension
HCC: Hepatocellular carcinoma
HE: Hepatic encephalopathy
MELD: Model of Endstage Liver Diseases
OS: Overall survival
PH: Portal hypertension
PVTT: Portal vein tumour thrombosis
RCT: Randomized controlled trial
TACE: Transarterial chemoembolization
TAE: Transcatheter arterial embolization
TKI: Tyrosine kinase inhibitor
